# Treatment-associated remodeling of the pancreatic cancer endothelium at single-cell resolution

**DOI:** 10.3389/fonc.2022.929950

**Published:** 2022-09-16

**Authors:** Carina Shiau, Jennifer Su, Jimmy A. Guo, Theodore S. Hong, Jennifer Y. Wo, Karthik A. Jagadeesh, William L. Hwang

**Affiliations:** ^1^ Center for Systems Biology, Massachusetts General Hospital, Harvard Medical School, Boston, MA, United States; ^2^ Department of Radiation Oncology, Massachusetts General Hospital, Harvard Medical School, Boston, MA, United States; ^3^ Center for Cancer Research, Massachusetts General Hospital, Harvard Medical School, Boston, MA, United States; ^4^ Broad Institute of Massachusetts Institute of Technology (MIT) and Harvard, Cambridge, MA, United States; ^5^ Biological and Biomedical Sciences Program, Harvard Medical School, Boston, MA, United States

**Keywords:** pancreatic cancer, endothelial cells, single-cell transcriptomics, radiotherapy – chemotherapy, losartan

## Abstract

Pancreatic ductal adenocarcinoma (PDAC) is one of the most treatment refractory and lethal malignancies. The diversity of endothelial cell (EC) lineages in the tumor microenvironment (TME) impacts the efficacy of antineoplastic therapies, which in turn remodel EC states and distributions. Here, we present a single-cell resolution framework of diverse EC lineages in the PDAC TME in the context of neoadjuvant chemotherapy, radiotherapy, and losartan. We analyzed a custom single-nucleus RNA-seq dataset derived from 37 primary PDAC specimens (18 untreated, 14 neoadjuvant FOLFIRINOX + chemoradiotherapy, 5 neoadjuvant FOLFIRINOX + chemoradiotherapy + losartan). A single-nucleus transcriptome analysis of 15,185 EC profiles revealed two state programs (ribosomal, cycling), four lineage programs (capillary, arterial, venous, lymphatic), and one program that did not overlap significantly with prior signatures but was enriched in pathways involved in vasculogenesis, stem-like state, response to wounding and hypoxia, and endothelial-to-mesenchymal transition (reactive EndMT). A bulk transcriptome analysis of two independent cohorts (*n* = 269 patients) revealed that the lymphatic and reactive EndMT lineage programs were significantly associated with poor clinical outcomes. While losartan and proton therapy were associated with reduced lymphatic ECs, these therapies also correlated with an increase in reactive EndMT. Thus, the development and inclusion of EndMT-inhibiting drugs (e.g., nintedanib) to a neoadjuvant chemoradiotherapy regimen featuring losartan and/or proton therapy may be most effective in depleting both lymphatic and reactive EndMT populations and potentially improving patient outcomes.

## Introduction

Pancreatic ductal adenocarcinoma (PDAC) is a treatment refractory and lethal malignancy that is projected to be the second leading cause of cancer mortality in the United States by 2030 (1). The tumor endothelium is a dynamic regulator of metabolism, oxygenation, angiogenesis, vasculogenesis, drug delivery, and metastasis, which is reflected in the diversity of intratumoral endothelial cell (EC) lineages (2). The properties and proportions of EC lineages in the tumor microenvironment impact the efficacy of antineoplastic therapies (3), which in turn remodel EC states and distributions (4–6). However, the treatment-associated remodeling and prognostic impact of EC lineages in the PDAC endothelium are poorly understood. Thus, it is paramount to develop a high-resolution understanding of the reciprocal influences between EC lineages and therapeutic interventions, which may guide novel therapeutic strategies to improve patient outcomes.

## Materials and methods

### Analysis of single-nucleus RNA-seq data

We extracted single-nucleus gene expression data from high-quality ECs using a custom subset of primary PDAC patients (DUOS dataset ID 000139) who underwent surgery without neoadjuvant therapy (*n* = 18), with neoadjuvant FOLFIRINOX + chemoradiation (*n* = 14), or with neoadjuvant FOLFIRINOX + chemoradiation + losartan (*n* = 5) as previously described (7). EC profiles were identified using gene markers for general endothelial: *PECAM1* and *VWF*; vascular endothelial: *ESAM*, *FLT1*, and *EPAS1*; and lymphatic endothelial: *FLT4*, *SEMA3A*, and *SEMA3D.* We applied non-negative matrix factorization (NMF) implemented in sklearn to decompose the gene expression matrix into two matrices, one of which embeds the endothelial gene expression programs. Because the result of NMF optimization can vary between runs based on random seeding, we repeated the NMF 50 times and computed a set of consensus programs by aggregating results from all 50 runs and determining the stability and reconstruction error. This consensus NMF was performed by making custom updates to the cNMF Python package. To determine the optimal number of programs *k*, we struck a balance between maximizing stability and minimizing error of the cNMF solution, while ensuring that the resulting programs were as biologically coherent and parsimonious as possible.

Each program was annotated by its top 200 weighted genes, utilizing a combination of gene set enrichment analysis (GSEA) and comparison with previously characterized endothelial signatures. Based on these annotations, each program was additionally classified as either a state or lineage program. To measure the similarity between PDAC-derived gene expression lineage programs and prior lung-derived single-cell endothelial lineage signatures (2), we performed the two-sided hypergeometric test. For analyses requiring lineage program assignments at the single-nucleus level, we classified each nucleus by its top-weighted cNMF lineage program. Similarly, for analyses requiring state program assignments, we classified each nucleus by its top-weighted cNMF state program.

For each endothelial program, a differential gene expression analysis using a mixed effects Poisson model was performed between cells classified as a given program and other ECs to identify enriched and depleted genes. We constructed the mixed effects model with the sample ID as a random effect; treatment status, three principal components, and sex as fixed effect covariates; and the log-normalized total unique molecular identifiers (UMIs) as an offset. The mixed effects model was implemented using the glmer R package.

We stratified neoadjuvant-treated patients (*n* = 19) into subgroups with (CRTL, *n* = 5) and without losartan (CRT, *n* = 14) and separately into subgroups receiving low-dose radiotherapy (31–39 GyE, *n* = 11) versus those receiving high-dose photon radiotherapy (55–59 GyE, *n* = 8). We further stratified the subset of low-dose radiotherapy-treated patients into subgroups with low-dose photon radiotherapy (*n* = 7) versus those with low-dose proton radiotherapy (*n* = 4). No patients in our cohort received high-dose proton radiotherapy. Two-sided Mann–Whitney U test with Benjamini–Hochberg correction (FDR = 0.1) was used to compare treatment status with the proportion of ECs classified as a given program as well as the average expression of a given gene across patients.

We explored the association among endothelial lineage programs with diverse stromal cell types (B, CD4^+^ T, CD8^+^ T, Tregs, dendritic, macrophages, mast, natural killer, neutrophils, and plasma) and previously described (7) malignant and cancer-associated fibroblast programs. For a given EC lineage program, a two-sided Mann–Whitney U test with Benjamini–Hochberg correction (FDR = 0.1) was used to compare the mean malignant/fibroblast program expression for patients in the top quartile versus patients in the bottom quartile of the EC lineage program expression. The same statistical test was used to compare the stromal-to-EC ratio for patients in the top quartile versus patients in the bottom quartile of the EC lineage program expression.

### Analysis of targeted transcriptome data

Targeted transcriptome data from cultured primary human umbilical vein endothelial cells (HUVECs) were obtained from Morilla et al (8). HUVECs were divided into groups: irradiated (*n* = 24) and non-irradiated (*n* = 24) conditions. To compute endothelial cNMF lineage program scores, we averaged the targeted transcriptome expression of genes overlapping with the top 200 weighted genes for each lineage program. Two-sided Wilcoxon signed-rank test with Bonferroni correction was used to compare lineage program expression scores across treatment conditions.

### Analysis of bulk RNA-seq data

Bulk RNA-seq data from two previously published resected untreated primary PDAC cohorts with clinical annotation were obtained (The Cancer Genome Atlas (9), *n* = 140; PanCuRx (10, 11), *n* = 168). Patients with metastases were excluded from this analysis. Gene expression levels from RNA-seq data were estimated using RSEM. We then deconvolved the cell-type proportions in each tumor using the following marker genes: endothelial (*PECAM1*, *VWF*), epithelial/acinar (*CFTR*, *KRT19*, *KRT7*, *KRT17*, *EPCAM*, *CEACAM6*, *COL17A1*, *MECOM*, *CPB1*, *PRSS3*, *AMY1A*), myeloid (*CD68*, *CD163*, *MRC1*, *CD80*, *CD86*, *TGFB1*, *CSM1*, *XCR1*, *CST3*, *CLEC9A*, *LGALS2*, *CD1A*, *CD207*, *CD1E*, *FCER1A*, *NDRG2*, *FSCN1*, *LAMP3*, *CCL19*, *CCR7*, *IRF7*, *LILRA4*, *TCF4*, *CXCR3*, *IRF4*, *CSF3R*, *CXCL8*), lymphoid (*CD4*, *CD8A*, *CD8B*, *CD3D*, *THEMIS*, *CD96*, *KZF1*, *GZMA*, *FOXP3*, *BANK1*, *CD19*, *KLRD1*, *KIR2DL3*, *IL18R1*, *KIR2DL1*, *KIR3DL2*, *SDC1*, *IGLC2*), cancer-associated fibroblast (*COL1A1*, *FN1*, *PDPN*, *DCN*, *VIM*, *FAP*, *ACTA2*, *IL6*, *C3*, *LIF*, *POSTN*, *FBLN1*), pericyte (*PDGFRB*, *DLK1*, *ACTA1*, *RGS5*, *CSPG4*, *MCAM*), Schwann (*SOX10*, *S100B*, *NGFR*), endocrine (*GCG*, *INS*, *APP*, *SST*, *PPY*, *GHRL*, *SYP*, *CHGA*, *VGF*), intra-pancreatic neurons (*TH*, *CHAT*, *ENO2*, *TAC1*), and adipocytes (*PLIN1*, *LPL*). To compute cNMF endothelial program scores, we summed the endothelial compartment expression of the top 200 weighted genes for each program, and the z-score normalized the expression scores within the TCGA (9) and PanCuRx (10, 11) cohorts independently to account for batch effects.

Age, sex, grade, stage, time to progression (TTP), and overall survival (OS) were available for 269 patients, of whom 154 had progression events and 167 died during follow-up. Multivariable Cox regression analysis was performed for TTP and OS with age, sex, grade, stage, and EC lineage program z-scores as covariates. Separately, the same analysis was performed with EC state program z-scores, instead of EC lineage program z-scores, as covariates.

Histopathologically annotated lymph node (N) staging was available for 279 patients (9–11). Patients were divided into two groups: those with (N^+^, *n* = 210) and without (N^-^, *n* = 69) lymph node metastasis. Two-sided Mann–Whitney U test was used to compare N staging with z-score-normalized lineage program expression scores.

## Results

### Single-nucleus RNA-seq captures diverse pancreatic EC states and lineages

**Figure 1:**
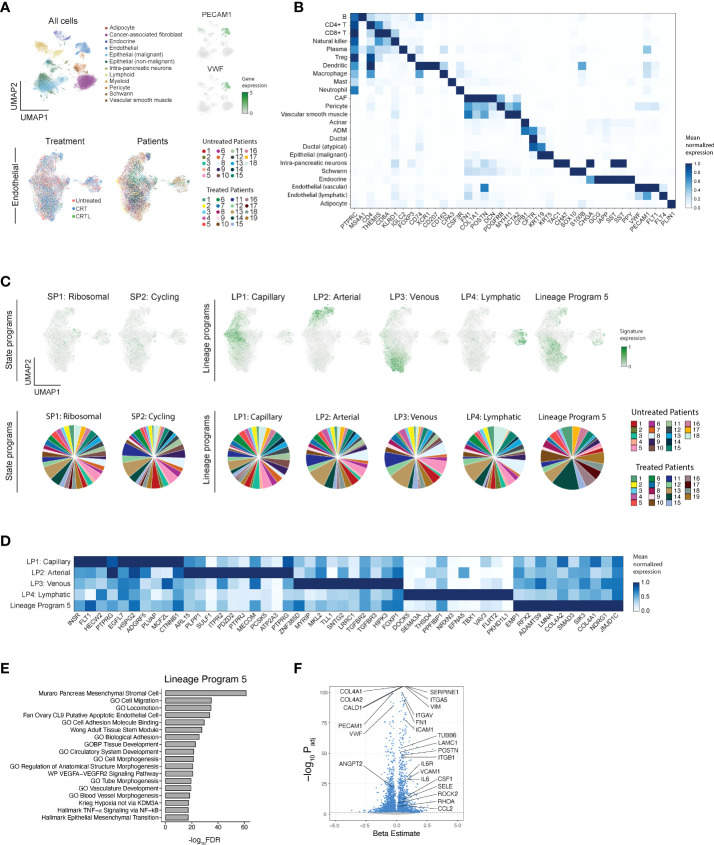
Single-nucleus RNA-seq of untreated and treated PDAC specimens captures the diversity of endothelial lineage programs including a reactive endothelial-to-mesenchymal transition (EndMT) program. **(A)** Single-nucleus RNA-seq captures diverse epithelial, immune, and stromal cell subsets. Top: UMAP embeddings of single-nucleus profiles (dots) of cells from all PDAC tumors, colored by annotated cell subsets (left) and general endothelial marker gene expression (right, insets). Bottom: Distinctions between treatment status and patient ID within the endothelial cell compartment. UMAP embeddings of single-nucleus profiles of endothelial cells, colored by treatment status (left; untreated, *n* = 18; CRT, *n* = 14; CRTL, *n* = 5) and patient ID (right). **(B)** Mean expression (color bar) of selected marker genes (columns) across annotated cell subsets (rows) across all PDAC tumors, normalized for each marker gene. **(C)** Top: UMAP embeddings of single-nucleus profiles (dots) of endothelial cells from all PDAC tumors (*n* = 37), colored by the normalized expression score of each of two state programs (left) and five lineage programs (right). Bottom: Proportion of nuclei in each of two state programs (left) and five lineage programs (right), colored by patient ID. **(D)** Mean expression (color bar) of the top 10 genes (columns) that characterize the endothelial lineage programs (rows), normalized for each gene. **(E)** Gene set enrichment analysis for Lineage Program 5. **(F)** Differential expression (beta estimate, x-axis; mixed effects model) and its significance (-log_10_(p_adj_ value), y-axis) for Lineage Program 5 endothelial cells *vs*. other endothelial cells. Selected enriched (positive beta estimate) and depleted (negative beta estimate) genes are labeled. Bonferroni-adjusted p value <0.05 is indicated with a dashed horizontal line. Genes with a significant Bonferroni-adjusted p value are colored blue and others are colored gray. SP, State Program; LP, Lineage Program.

We extracted 15,185 high-quality EC profiles by applying known cell-type signatures (2) to a custom single-nucleus RNA-seq dataset (7) derived from 37 patients with primary PDAC who underwent surgical resection with (*n* = 19) or without neoadjuvant treatment (*n* = 18) (**Figures 1A, B**; **Supplementary Figure 1A**). Neoadjuvant treatment consisted of multicycle chemotherapy (FOLFIRINOX) and consolidative chemoradiation (5-FU or capecitabine) with (CRTL; *n* = 5) or without (CRT; *n* = 14) losartan (12).

To learn recurrent *de novo* expression programs in an unbiased manner across all intratumoral ECs, we performed consensus non-negative matrix factorization (cNMF). We selected the number of programs (*k* = 9) based on optimizing stability and error (**Figure 1C**; **Supplementary Figure 1B**; Methods) and focused on programs that were well-distributed across ECs from multiple patients (**Figure 1C** bottom). Programs were annotated by their top 200 weighted genes based on gene set enrichment analysis (GSEA) and similarity to previously identified endothelial subtypes (2), yielding two cell state programs (ribosomal, cycling); four cell lineage programs (capillary, arterial, venous, lymphatic); and one program (Lineage Program 5) that did not overlap significantly with prior endothelial signatures (**Figures 1C-F**; **Supplementary Figures 1C-E**; Methods). EC signatures identified by Schupp et al. (2) overlapped strongly with four EC cNMF programs: Lineage Program 1 (capillary) [Schupp general capillary (p = 8.95 × 10^-2^; two-sided hypergeometric) and general capillary-aerocyte (p = 4.20 × 10^-9^)], Lineage Program 2 (arterial) [Schupp arterial (p = 2.37 × 10^-18^) and arterial-venous (p = 2.32 × 10^-2^)], Lineage Program 3 (venous) [Schupp general venous (p = 1.16 × 10^–2^), systemic venous (p = 2.02 × 10^-9^), pulmonary venous (p = 8.21 × 10^-6^), and arterial-venous (p = 1.35 × 10^-4^)], and Lineage Program 4 (lymphatic) [Schupp lymphatic (p = 1.69 × 10^-18^)] (**Figure 1C** **;** **Supplementary Figures 1D, E** **)**.

### A reactive endothelial-to-mesenchymal transition (EndMT) lineage program is enriched in neoadjuvant-treated pancreatic tumor specimens and irradiated HUVECs

**Figure 2:**
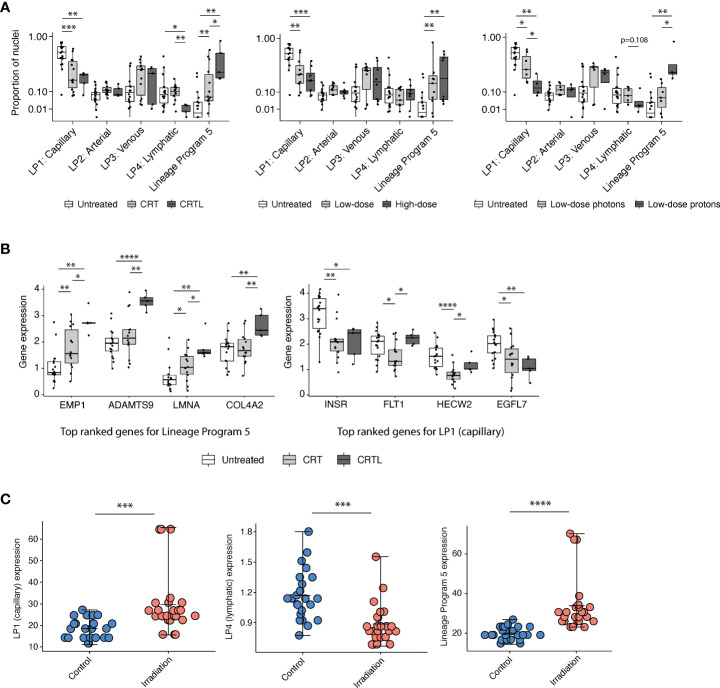
Capillary program depletion and reactive EndMT program enrichment in post-treatment residual human tumor specimens. **(A)** Proportion of endothelial nuclei for each of the five lineage programs from PDAC tumors stratified by treatment status (left; untreated, *n* = 18; CRT, *n =* 14; CRTL, *n* = 5), radiation dose (middle; untreated, *n* = 18; low-dose, *n* = 11; high-dose, *n* = 8), and radiation type (right; untreated, *n* = 18; low-dose photons, *n* = 7; low-dose protons, *n* = 4). * Benjamini–Hochberg-adjusted p value < 0.05, **p < 0.01, ***p < 0.001, FDR = 0.1, and two-sided Mann–Whitney U test. **(B)** Mean expression of top-ranked genes that characterize Lineage Program 5 (left) and Lineage Program 1 (capillary) (right) in endothelial nuclei across all PDAC samples, separated by treatment status (untreated, *n* = 18; CRT, *n* = 14; CRTL, *n* = 5). *Benjamini–Hochberg-adjusted p value < 0.05, **p < 0.01, ****p < 0.0001, FDR = 0.1, and two-sided Mann–Whitney U test. **(C)** Lineage Program 1 (capillary; Bonferroni-adjusted p value = 1.33 × 10^-4^; two-sided Wilcoxon signed-rank test), Lineage Program 4 (lymphatic; p = 6.15 × 10^-4^), and Lineage Program 5 (p = 6.79 × 10^-6^) expression in non-irradiated versus irradiated primary human umbilical vein endothelial cells *in vitro (* 8) (control, *n =* 24; irradiation, *n =* 24). Mean expression is denoted by the central hash and error bars extend from the minimum to maximum values for each condition. ***Bonferroni-adjusted p value <0.001, ****p < 0.0001, two-sided Wilcoxon signed-rank test. LP, Lineage Program.

Lineage Program 5 is enriched for pathways involved in vasculogenesis, stem-like and mesenchymal states, and response to wounding and hypoxia (**Figures 1E, F** **)**. Treatment was associated with a higher proportion of Lineage Program 5 endothelial nuclei (CRT *vs*. untreated, p = 3.82 × 10^-3^; CRTL *vs*. untreated, p = 1.86 × 10^-3^; CRTL *vs*. CRT, p = 4.65 × 10^-2^; two-sided Mann–Whitney U test) and a lower proportion of Lineage Program 1 (capillary) endothelial nuclei (CRT *vs*. untreated, p = 8.88 × 10^-4^; CRTL *vs*. untreated, p = 2.54 × 10^-3^) (**Figure 2A**). At the individual gene level, we observed similar trends when we examined the top-ranked genes that characterize Lineage Program 5 and Lineage Program 1 (capillary) (**Figure 2B**). With the addition of losartan, we observed a lower proportion of Lineage Program 4 (lymphatic) endothelial nuclei (CRTL *vs*. untreated, p = 2.30 × 10^-2^; CRTL *vs*. CRT, p = 3.54 × 10^-3^) (**Figure 2A**).

Radiation has dose-dependent effects on the vasculature (13). Proton therapy is endowed with dose distribution and radiobiologic advantages compared with photon therapy that may lead to clinical benefit in certain contexts (14). To assess whether endothelial lineage program expression is associated with radiation dose and modality, we stratified neoadjuvant-treated patients into a low-dose mixed modality radiotherapy subgroup with a biologically effective dose (BED) of 31–39 Gy equivalents (GyE) (*n* = 11) versus a high-dose photon radiotherapy subgroup (*n* = 8) with a BED of 55–59 Gy and separately into low-dose photon (*n* = 7) versus low-dose proton (*n* = 4) subgroups (**Figure 2A**). We observed that neoadjuvant treatment that includes radiotherapy, especially proton therapy, was associated with a lower proportion of Lineage Program 1 (capillary) endothelial nuclei (high-dose photon *vs*. untreated, p = 6.34 × 10^-4^; low-dose proton *vs*. untreated, p = 5.67 × 10^-3^; low-dose proton *vs*. low-dose photon, p = 4.72 × 10^-2^), consistent with prior observations that radiation-induced endothelial damage is concentrated in the microvasculature (15) rather than larger vessels (**Figure 2A**). Furthermore, high-dose photon radiotherapy (high-dose *vs*. untreated, p = 1.99 × 10^-3^) and low-dose proton therapy (low-dose proton *vs*. untreated, p = 3.14 × 10^-3^; low-dose proton *vs*. low-dose photon, p = 1.07 × 10^-2^) were both associated with a higher proportion of Lineage Program 5 endothelial nuclei (**Figure 2A**). The lack of significant differences in lineage program proportions between our high- and low-dose radiotherapy groups may be secondary (1) to all high-dose treatments that used photons *vs*. 36% of low-dose treatments that used protons; (2) for vascular effects, in which high-dose radiation is typically defined as greater than 10 GyE (13) so both the low- and high-dose groups in our dataset are well above this threshold; and/or (3) to insufficient statistical power.

To further explore the biological features of Lineage Program 5, we performed a differential expression (DE) analysis of Lineage Program 5 ECs versus all other EC subtypes (**Figure 1E, F** **)**. We observed that Lineage Program 5 endothelial nuclei were enriched in leukocyte adhesion molecules (*SELE*/E-selectin, *VCAM1*, *ICAM1*), which when combined with enrichment in HALLMARK_TNFA_SIGNALING_VIA_NFKB pathway genes suggests an inflammatory role for Lineage Program 5 (**Figure 1E, F**; Methods). Furthermore, consistent with prior studies demonstrating that moderate and high doses (>2 GyE) of radiation inhibit angiogenesis, the DE analysis revealed downregulation of *VWF* and *ANGPT2* and concomitant enrichment of *IL6* and *IL6R (* 13) (**Figure 1F**). In lieu of local angiogenesis, new vessel formation to support tumor recurrence after moderate- and high-dose radiation is dependent on vasculogenesis from bone marrow-derived cells (13). Lineage Program 5 ECs are enriched in *CSM1* and *CCL2*, two factors that can increase the recruitment of tumor-associated macrophages that facilitate vasculogenesis (13). Radiation also enhances microvascular permeability in a dose-dependent manner, in part mediated by RhoA and Rho-associated kinases that regulate actin cytoskeletal organization and modulate the integrity of cell–cell junctions; the Lineage Program 5 differential expression analysis demonstrated an enrichment in both *RHOA* and *ROCK2* (**Figure 1F**). Moreover, Lineage Program 5 exhibits downregulation of characteristic endothelial markers (e.g., *PECAM1*, *VWF*) and enrichment for mesenchymal markers (e.g., *VIM*, *FN1*, *POSTN*, *COL4A1*, *COL4A2*) (**Figure 1F**), suggestive of an endothelial-to-mesenchymal transition (EndMT) phenotype that is similar to epithelial–mesenchymal transition (EMT) and associated with abnormal pericyte recruitment, vasculogenesis, proliferation of radioresistant cancer cells with stem-like properties, and promotion of radiation-induced tissue fibrosis (16). In the context of treatment-associated enrichment (**Figure 2A**), these biological features of Lineage Program 5 suggest that it may represent a reactive EndMT phenotype.

We further validated the identification of a treatment-associated reactive EndMT phenotype by comparing Lineage Program 5 expression in non-irradiated (*n* = 24) versus irradiated (*n* = 24) primary human umbilical vein endothelial cells (HUVECs) with targeted transcriptome data (8). Lineage Program 5 (reactive EndMT) expression is significantly enriched in irradiated HUVECs *in vitro* (Bonferroni-adjusted p value = 6.79 × 10^-6^; two-sided Wilcoxon signed-rank test) (**Figure 2C**). The expression of Lineage Program 4 (lymphatic) was also significantly depleted in irradiated HUVECs *in vitro* (p = 6.15 × 10^-4^) (**Figure 2C**), which is consistent with our findings that the use of losartan and proton therapy is associated or borderline associated with a decrease in Lineage Program 4 (lymphatic) expression (**Figure 2A**). However, the expression of Lineage Program 1 (capillary) was significantly enriched in irradiated HUVECs *in vitro* (**Figure 2C**), which is different from our findings that Lineage Program 1 (capillary) is depleted after treatment in patient tumors (**Figure 2A**). The discrepancy in these findings could result from differences in treatment regimens received by patients in the snRNA-seq dataset (chemotherapy and radiotherapy) (7) versus HUVECs in the targeted transcriptome dataset (radiotherapy only) (8), the former is not matched across the treated and untreated conditions, and the latter lacks the context of a tumor microenvironment.

**Figure 3:**
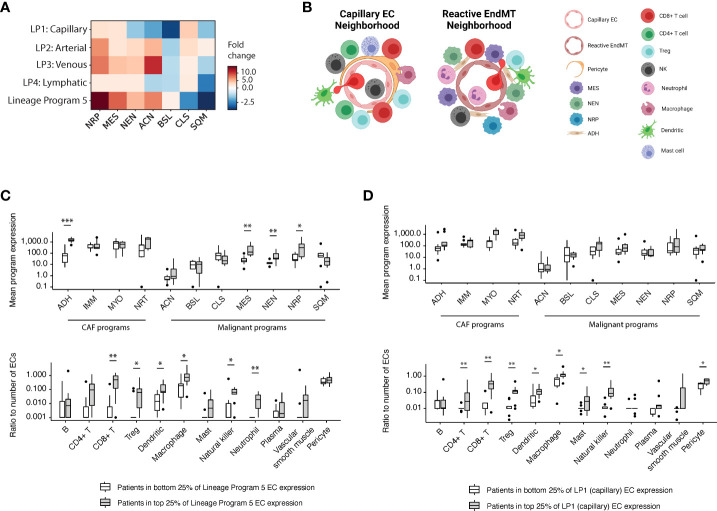
Reactive EndMT endothelial cells are associated with malignant cells expressing neural-like progenitor, mesenchymal, and neuroendocrine-like programs. **(A)** Fold change (color bar) of malignant programs (columns) between top quartile versus bottom quartile scoring patients for endothelial lineage programs (rows). **(B)** Schematic of enriched malignant programs, cancer-associated fibroblast (CAF) programs, and diverse stromal/immune cell type populations in the tumor microenvironment surrounding Lineage Programs 1 (capillary; left) and 5 (reactive EndMT; right) endothelial cells. **(C, D)** Top: Mean expression of CAF and malignant programs between top quartile versus bottom quartile scoring patients for Lineage Program 5 **(C)** and Lineage Program 1 (capillary) **(D)** EC expression in the snRNA-seq data (7). Bottom: Ratio of the number of stromal/immune cell type (x axis) to endothelial cells between top quartile versus bottom quartile scoring patients for Lineage Program 5 **(C)** and Lineage Program 1 (capillary) **(D)** endothelial nuclei expression. *Benjamini–Hochberg-adjusted p value < 0.05, **p < 0.01, ***p < 0.001, FDR = 0.1, and two-sided Mann–Whitney U test. CAF programs: ADH, adhesive; IMM, immunomodulatory; MYO, myofibroblast; NRT, neurotropic. Malignant programs: ACN, acinar; BSL, basaloid; CLS, classical-like; MES, mesenchymal; NEN, neuroendocrine-like; NRP, neural-like progenitor; SQM, squamoid. LP, Lineage Program.

To explore the tumor microenvironment associated with the endothelial lineage programs we identified, we examined the snRNA-seq dataset (7) to investigate whether there was an association among endothelial lineage programs and previously identified malignant/fibroblast programs (7). We observed that malignant cells from patients in the top quartile of Lineage Program 5 EC expression had more than a 14-, 8-, and 4-fold enrichment in neural-like progenitor (NRP, p = 2.17 × 10^-2^; two-sided Mann–Whitney U test), mesenchymal (MES, p = 2.68 × 10^-3^), and neuroendocrine-like (NEN, p = 6.19 × 10^-3^) program expression, respectively, compared with those of patients in the bottom quartile of Lineage Program 5 EC expression (**Figures 3A-C**). Notably, in our prior work, the NRP and NEN programs were significantly enriched in post-treatment specimens, the NRP program was additionally associated with poor prognosis, and the MES program trended toward enrichment after treatment (7). The latter aligns with prior studies in which an epithelial-to-mesenchymal (EMT) phenotype in PDAC was linked to treatment resistance, shorter survival, tumor metastasis, and disease progression (17, 18).

Exploring potential associations among Lineage Program 5 ECs and diverse stromal and immune cell types (B, CD4^+^ T, CD8^+^ T, Tregs, dendritic, macrophages, mast, natural killer, neutrophils, plasma, vascular smooth muscle, and pericytes), we observed that the ratios of CD8^+^ T cells (p = 6.08 × 10^-3^, two-sided Mann–Whitney U test), dendritic cells (p = 2.16 × 10^-2^), macrophages (p = 2.17 × 10^-2^), natural killer cells (p = 2.76 × 10^-2^), neutrophils (p = 1.85 × 10^-3^), and Tregs (p = 1.40 × 10^-2^) to endothelial cells were enriched in patients with a higher expression of Lineage Program 5 ECs (**Figure 3B, C** **)**. The high expression of macrophage chemoattractants by Lineage Program 5 endothelial cells such as *CSM1* and *CCL2* (**Figure 1F**) may be driving the higher macrophage-to-endothelial ratio in Lineage Program 5–enriched patients (**Figure 3C**). Additionally, we observed that the ratios of CD8^+^ T cells (p = 1.32 × 10^-3^, two-sided Mann–Whitney U test), CD4^+^ T cells (p = 3.00 × 10^-3^), natural killer cells (p = 4.30 × 10^-3^), Tregs (p = 5.67 × 10^-3^), macrophages (p = 2.17 × 10^-2^), dendritic cells (p = 3.40 × 10^-2^), mast cells (p = 3.45 × 10^-2^), and pericytes (p = 3.41 × 10^-2^) to endothelial cells were enriched in tumors that had a higher expression of Lineage Program 1 (capillary) ECs (**Figures 3B, D** **)**. We note, however, that both of these analyses were conducted at an aggregated patient level since we lack spatial information in the snRNA-seq dataset (7).

We chose in part to focus our analysis on the endothelial lineage programs rather than the state programs because State Program 1 (ribosomal) and State Program 2 (cycling) do not have significant associations with treatment (**Supplementary Figure 2A**). Moreover, we recognized that treated patients 13 and 14 constituted a large proportion of the EC population (**Supplementary Figure 1A**) and particularly of the Lineage Program 5 subgroup (**Figure 1C**). Thus, we investigated whether the treatment associations (**Figure 2A**) that we observed were robust to the removal of treated patients 13 and 14, and we found that the results were largely consistent (**Supplementary Figure 2B**). Treatment remained associated with a higher proportion of Lineage Program 5 endothelial nuclei and a lower proportion of Lineage Program 1 (capillary) endothelial nuclei, while the addition of losartan was still associated with a lower proportion of Lineage Program 4 (lymphatic) endothelial nuclei (**Supplementary Figure 2B**). We also found that high-dose photon radiotherapy and low-dose proton therapy were still associated with a higher proportion of Lineage Program 5 endothelial nuclei, whereas high-dose photon radiotherapy was associated with a lower proportion of Lineage Program 1 (capillary) endothelial nuclei (**Supplementary Figure 2B**). The only difference relative to the full 37-patient dataset is that we no longer observed a significant depletion in the proportion of Lineage Program 1 (capillary) endothelial nuclei after low-dose proton radiotherapy, although the trend remained apparent (**Supplementary Figure 2B**). This consistency with subsampling indicates that the endothelial profiles from treated patients 13 and 14 are not unduly influencing our results.

### Lymphatic and reactive EndMT expression portend poor prognosis in untreated patients

**Figure 4:**
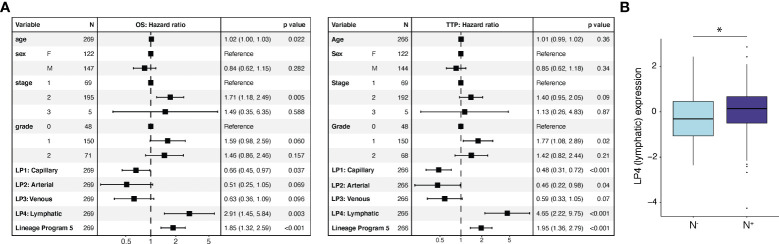
Lymphatic and reactive EndMT programs are associated with poor prognosis in independent bulk RNA-seq cohorts. **(A)** Endothelial lineage program expression and clinicopathological parameters associated with overall survival (OS; left) and time to progression (TTP; right) using a multivariable Cox regression analysis. *p value < 0.05, two-sided Mann-Whitney U test. **(B)** Lineage Program 4 (lymphatic) association with pathologically annotated lymph node involvement (N^-^, *n* = 69; N^+^, *n* = 210; p = 2.67 × 10^-2^, two-sided Mann–Whitney U test). Cox regression **(A)** and lymph node association **(B)** analyses use deconvolved bulk RNA-seq data from two independent cohorts of untreated, resected primary PDAC specimens [TCGA (9) (*n* = 135) and PanCuRx (10, 11) (*n* = 134) for Cox regression; TCGA (*n* = 135) and PanCuRx (*n* = 144) for lymph-node association]. LP, Lineage Program.

To assess the prognostic relevance of these endothelial state and lineage programs, we scored them in clinically annotated bulk RNA-seq data (7) from two independent cohorts of patients with untreated, resected primary PDAC from TCGA (9) (*n* = 135) and PanCuRx (10, 11) (*n* = 134) (Methods). We deconvolved the cell-type proportions for each tumor and computed the z-score-normalized program scores for the endothelial compartment. We then performed a multivariable Cox regression analysis of the overall survival (OS) and time to progression (TTP) endpoints with age, sex, stage, grade, and the z-score-normalized endothelial program expression as covariates. While age, sex, stage, and grade were not prognostic for TTP, age and stage were prognostic for OS (**Figure 4A**). Lineage Program 4 (lymphatic) (OS: HR 2.91, 95% CI 1.45–5.84, p = 0.003; TTP: HR 4.65, 95% CI 2.22–9.75, p < 0.001) and Lineage Program 5 (OS: HR 1.85, 95% CI 1.32–2.59, p < 0.001; TTP: HR 1.95, 95% CI 1.36–2.79, p < 0.001) were associated with lower OS and shorter TTP. On the other hand, Lineage Program 1 (capillary) (OS: HR 0.66, 95% CI 0.45–0.97, p = 0.037; TTP: HR 0.48, 95% CI 0.31–0.72, p < 0.001) was associated with higher OS and longer TTP (**Figure 4A**), which may be related to capillary endothelial cells increasing intratumoral drug delivery and improving tumor oxygenation, which would act as a radiosensitizer, although functional validation of this hypothesis is needed.

Lineage Program 2 (arterial) (OS: HR 0.51, 95% CI 0.25–1.05, p = 0.069; TTP: HR 0.46, 95% CI 0.22–0.98, p = 0.04) and Lineage Program 3 (venous) (OS: HR 0.63, 95% CI 0.36–1.09, p = 0.096; TTP: HR 0.59, 95% CI 0.33–1.05, p = 0.07) trended toward a positive association for both OS and TTP but did not reach significance (**Figure 4A**). While State Program 2 (cycling) was associated with shorter TTP (HR 1.23, 95% CI: 1.00–1.50, p = 0.047), this association did not persist when we examined OS (**Supplementary Figure 2C**). We caution that applying snRNA-seq-derived programs to bulk profiles may be confounded by non-endothelial contaminating cell types that express some of the endothelial program genes at relatively high levels. However, we note that the EC lineage programs are most highly expressed in vascular and lymphatic endothelial cells rather than other non-endothelial cell populations (**Supplementary Figure 2D**).

Next, we extracted the lymph node status from surgical pathology (9–11). Specimens were either annotated as having spread to regional lymph nodes (N^+^; n = 210) or not (N^-^; n = 69). Node-positive patients had significantly higher Lineage Program 4 (lymphatic) EC expression in the primary tumor (p = 2.67 × 10^-2^; two-sided Mann–Whitney U test), suggesting that lymph node involvement can potentially be predicted from the prevalence of Lineage Program 4 (lymphatic) ECs in the primary tumor (**Figure 4B**).

Interestingly, the use of losartan (CRTL *vs*. untreated, p = 2.30 × 10^-2^; CRTL *vs*. CRT, p = 3.54 × 10^-3^) and/or proton therapy (low-dose proton *vs*. low-dose photon, p = 1.08 × 10^-1^) was associated or borderline associated with lower Lineage Program 4 (lymphatic) EC expression (**Figure 2A**). Prior studies have demonstrated that genes involved in lymphangiogenesis were downregulated after losartan treatment (13) and proton radiation (4), which may help explain the association between these therapies and lower lymphatic program expression.

## Discussion

In summary, we extracted 15,185 high-quality EC profiles from a custom single-nucleus RNA-seq dataset (7) derived from 37 patients with primary PDAC who underwent surgical resection with (*n* = 19) or without neoadjuvant treatment (*n* = 18). Performing consensus non-negative matrix factorization (cNMF), we learned seven endothelial expression programs: two cell state programs (ribosomal, cycling), four cell lineage programs (capillary, arterial, venous, lymphatic), and one program (Lineage Program 5) that did not significantly overlap with prior endothelial subtype signatures (2). Lineage Program 5 was associated with the downregulation of characteristic endothelial markers and the enrichment of inflammatory and mesenchymal markers compared with other endothelial programs, suggesting a reactive endothelial-to-mesenchymal transition (EndMT) phenotype.

We discovered that higher proportions of Lineage Program 4 (lymphatic) and Lineage Program 5 (reactive EndMT) EC expression portended a poor prognosis in patients with untreated primary resected PDAC (9–11). While both proton therapy and losartan were associated with lower Lineage Program 4 (lymphatic) EC expression, these therapies also correlated with a higher prevalence of Lineage Program 5 (reactive EndMT) ECs. Interestingly, Lineage Program 5 (reactive EndMT) ECs were associated with malignant cells with a high expression of neural-like progenitor, neuroendocrine-like, and mesenchymal programs, which have been linked to treatment-resistant phenotypes with poor clinical outcomes (7, 14, 15). We further validated the treatment-associated enrichment of Lineage Program 5 (reactive EndMT) EC expression and depletion of Lineage Program 4 (lymphatic) EC expression in primary human umbilical vein endothelial cells (HUVECs) undergoing ionizing radiation *in vitro (* 8). Taken together, these results motivate further investigation into the potential collaborative interactions among Lineage Program 5 (reactive EndMT) ECs; neural-like progenitor, neuroendocrine-like, and mesenchymal malignant cells; and various immune cell types that may mediate therapeutic resistance in PDAC. From a clinical perspective, our study suggests that a combination of drugs with EndMT-inhibiting effects (16) (e.g., nintedanib) and a neoadjuvant chemoradiotherapy regimen featuring losartan and/or proton therapy may be worthwhile exploring.

## Data availability statement

Raw and processed human sequencing data (snRNA-seq) have been deposited in NCBI’s Gene Expression Omnibus and are accessible through GEO Series accession numbers GSE202051.

## Ethics statement

This study was reviewed and approved by Massachusetts General Hospital Institutional Review Board. The patients/participants provided their written informed consent to participate in this study.

## Author contributions

CS, JS, JG, KJ, and WH developed the study concept and designed the computational analyses. CS analyzed the data with guidance from KJ and WH, scientific insights from JS, JG, and WH, and clinical insights from TH and JW. CS generated the figures. WH supervised the research. CS and WH wrote the manuscript, and all authors reviewed the manuscript. All authors contributed to the article and approved the submitted version.

## Funding

This work was supported in part by NCI K08CA270417 (WH), Burroughs Wellcome Fund Career Award for Medical Scientists (WH), and Hopper Belmont Foundation Inspiration Award (WH). This study was also conducted with support of the Ontario Institute for Cancer Research (PanCuRx Translational Research Initiative) through funding provided by the Government of Ontario, the Wallace McCain Centre for Pancreatic Cancer supported by the Princess Margaret Cancer Foundation, the Terry Fox Research Institute, the Canadian Cancer Society Research Institute, and the Pancreatic Cancer Canada Foundation.

## Acknowledgments

We are grateful to our patients and their families, Aviv Regev, Tyler Jacks, Hannah I. Hoffman, Payman Yadollahpour, Cristina R. Ferrone, Motaz Qadan, Rakesh K. Jain, Ramnik Xavier, Andrew J. Aguirre, Mari Mino-Kenudson, Carlos Fernandez-del Castillo, Andrew S. Liss, David T. Ting, and many others for their contributions to the single-nucleus RNA-seq dataset.

## Conflict of interest

The authors declare that the research was conducted in the absence of any commercial or financial relationships that could be construed as a potential conflict of interest.

## Publisher’s note

All claims expressed in this article are solely those of the authors and do not necessarily represent those of their affiliated organizations, or those of the publisher, the editors and the reviewers. Any product that may be evaluated in this article, or claim that may be made by its manufacturer, is not guaranteed or endorsed by the publisher.

## Supplementary material

Supplementary Figure 1
**(A)** Distribution of endothelial nuclei across 37 treated and untreated PDAC samples. **(B)** Estimated stability (blue, left y axis) and error (red, right y axis) in the cNMF solution learned with different numbers of components/programs (*k*, x axis) for endothelial cells. **(C)** Gene set enrichment analyses (-log_10_ FDR) for State Program 1 (ribosomal; left) and State Program 2 (cycling; right). **(D)** Lineage program annotations based on similarity to prior endothelial cell signatures from Schupp et al (2). * p value < 0.05, ** p < 0.01, *** p < 0.001, two-sided hypergeometric test. **(E)** Mapping of single-cell RNA-seq lung endothelial cell profiles from Schupp et al (2) onto the PDAC endothelial cells in our snRNA-seq dataset. UMAP embeddings of single-nucleus profiles (dots) of endothelial cells, colored by the average gene expression (color bar) for each of the Schupp et al (2) signatures. SP = State Program; LP = Lineage Program.

Supplementary Figure 2
**(A)** Proportion of endothelial nuclei assigned to each of the two state programs for all PDAC tumors (*n*=37) stratified by treatment status (left; untreated, *n*=18; CRT, *n=*14; CRTL, *n*=5), radiation dose (middle; untreated, *n*=18; low-dose, *n*=11; high-dose, *n*=8), and radiation type (right; untreated, *n*=18; low-dose photons, *n*=7; low-dose protons, *n*=4). **(B)** Proportion of endothelial nuclei for each of the five lineage programs from a subset of PDAC tumors with treated patients 13 and 14 removed *(n*=35) stratified by treatment status (left; untreated, *n*=18; CRT, *n=*12; CRTL, *n*=5), radiation dose (middle; untreated, *n*=18; low-dose, *n*=10; high-dose, *n*=7), and radiation type (right; untreated, *n*=18; low-dose photons, *n*=5; low-dose protons, *n*=4). * Benjamini-Hochberg-adjusted p value < 0.05, ** p < 0.01, *** p < 0.001, FDR = 0.1 and two-sided Mann-Whitney U test. **(C)** Endothelial state program expression and clinicopathological parameters associated with overall survival (OS; left) and time to progression (TTP; right) using a multivariable Cox regression analysis on deconvolved bulk RNA-seq data from two independent cohorts of untreated, resected primary PDAC specimens [TCGA (9) (*n*=135) and PanCuRx (10, 11) (*n*=134)]. **(D)** Mean expression of endothelial lineage programs in various cell type populations in the single-nucleus RNA-sequencing dataset, normalized for each program. SP = State Program; LP = Lineage Program.
