# Supplementary figures and images for: Treatment-associated remodeling of the pancreatic cancer endothelium at single-cell resolution
Source: Front Oncol. 2022 Sep 16;12:929950. doi: 10.3389/fonc.2022.929950 (PMC9524152; doi:10.3389/fonc.2022.929950)

A

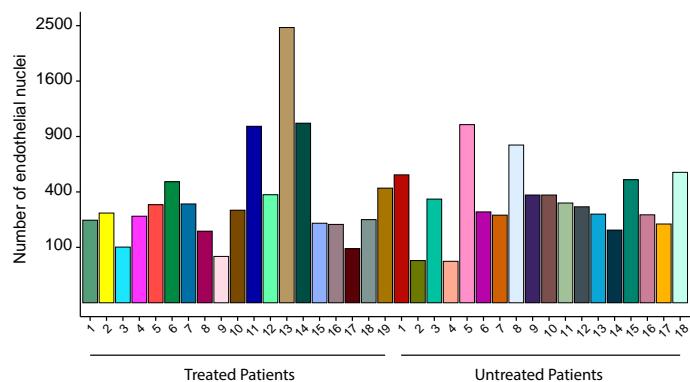

B

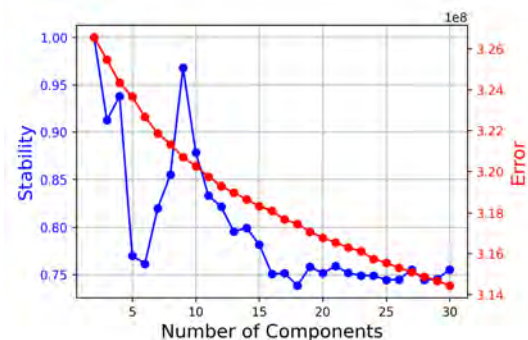

C

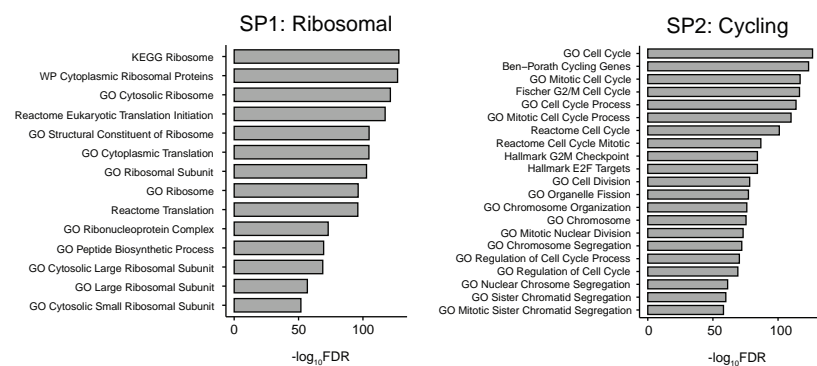

D

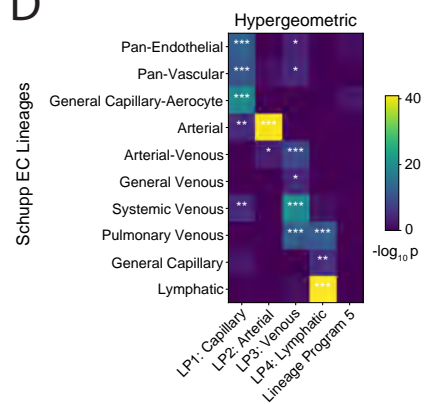

E

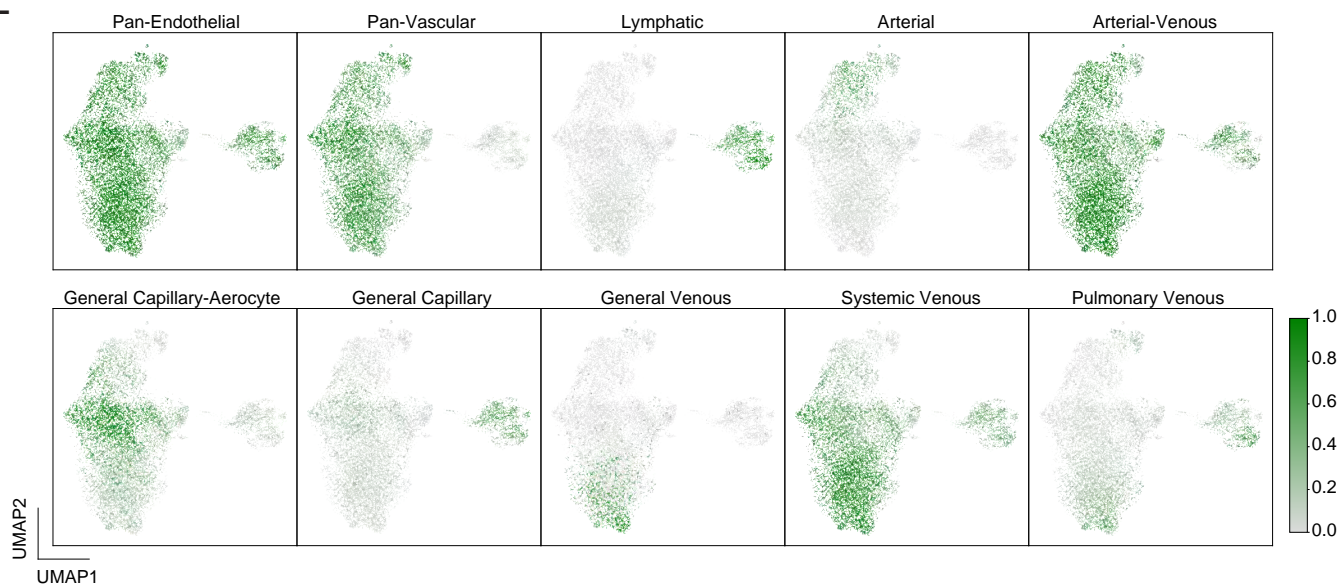

Supplement: Supplementary Figure 1 — (A) Distribution of endothelial nuclei across 37 treated and untreated PDAC samples. (B) Estimated stability (blue, left y axis) and error (red, right y axis) in the cNMF solution learned with different numbers of components/programs (k, x axis) for endothelial cells. (C) Gene set enrichment analyses (-log10 FDR) for State Program 1 (ribosomal; left) and State Program 2 (cycling; right). (D) Lineage program annotations based on similarity to prior endothelial cell signatures from Schupp et al (2). * p value < 0.05, ** p < 0.01, *** p < 0.001, two-sided hypergeometric test. (E) Mapping of single-cell RNA-seq lung endothelial cell profiles from Schupp et al (2) onto the PDAC endothelial cells in our snRNA-seq dataset. UMAP embeddings of single-nucleus profiles (dots) of endothelial cells, colored by the average gene expression (color bar) for each of the Schupp et al (2) signatures. SP = State Program; LP = Lineage Program. [file DataSheet_1.pdf]
