# Supplementary material for: Treatment-associated remodeling of the pancreatic cancer endothelium at single-cell resolution
Source: Front Oncol. 2022 Sep 16;12:929950. doi: 10.3389/fonc.2022.929950 (PMC9524152; doi:10.3389/fonc.2022.929950)
Supplement: Supplementary Figure 2 — (A) Proportion of endothelial nuclei assigned to each of the two state programs for all PDAC tumors (n=37) stratified by treatment status (left; untreated, n=18; CRT, n=14; CRTL, n=5), radiation dose (middle; untreated, n=18; low-dose, n=11; high-dose, n=8), and radiation type (right; untreated, n=18; low-dose photons, n=7; low-dose protons, n=4). (B) Proportion of endothelial nuclei for each of the five lineage programs from a subset of PDAC tumors with treated patients 13 and 14 removed (n=35) stratified by treatment status (left; untreated, n=18; CRT, n=12; CRTL, n=5), radiation dose (middle; untreated, n=18; low-dose, n=10; high-dose, n=7), and radiation type (right; untreated, n=18; low-dose photons, n=5; low-dose protons, n=4). * Benjamini-Hochberg-adjusted p value < 0.05, ** p < 0.01, *** p < 0.001, FDR = 0.1 and two-sided Mann-Whitney U test. (C) Endothelial state program expression and clinicopathological parameters associated with overall survival (OS; left) and time to progression (TTP; right) using a multivariable Cox regression analysis on deconvolved bulk RNA-seq data from two independent cohorts of untreated, resected primary PDAC specimens [TCGA (9) (n=135) and PanCuRx (10, 11) (n=134)]. (D) Mean expression of endothelial lineage programs in various cell type populations in the single-nucleus RNA-sequencing dataset, normalized for each program. SP = State Program; LP = Lineage Program. [file DataSheet_2.pdf]

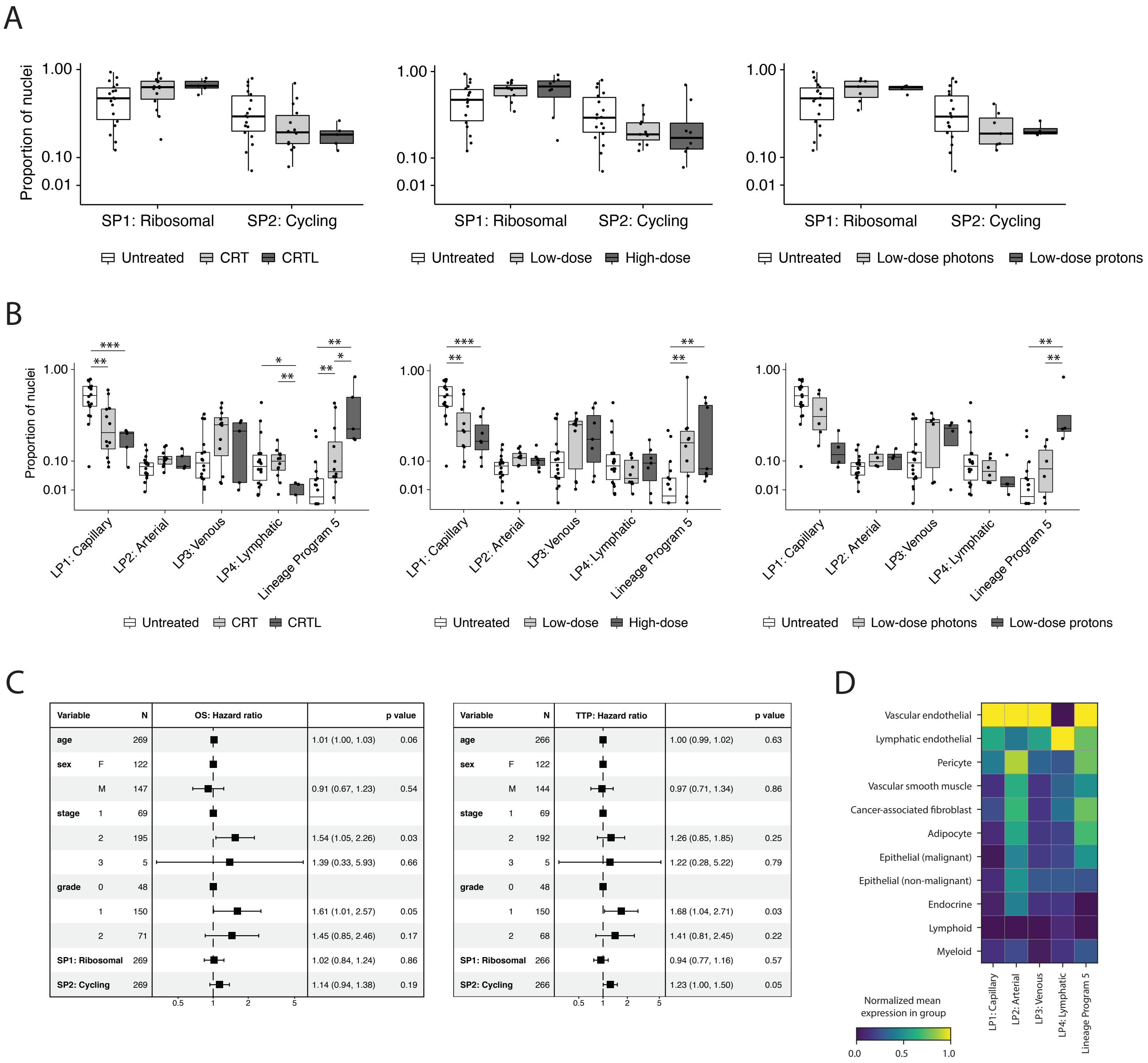
A
Proportion of nuclei
1.00
0.10
0.01
SP1: Ribosomal
SP2: Cycling
Untreated
CRT
CRTL
Low-dose
High-dose
Low-dose photons
Low-dose protons
B
Proportion of nuclei
LP1: Capillary
LP2: Arterial
LP3: Venous
LP4: Lymphatic
Lineage Program 5
***
**
*
C
Variable
N
OS: Hazard ratio
p value
age 269 1.01 (1.00, 1.03) 0.06
sex F 122
M 147 0.91 (0.67, 1.23) 0.54
stage 1 69
2 195 1.54 (1.05, 2.26) 0.03
3 5 1.39 (0.33, 5.93) 0.66
grade 0 48
1 150 1.61 (1.01, 2.57) 0.05
2 71 1.45 (0.85, 2.46) 0.17
SP1: Ribosomal 269 1.02 (0.84, 1.24) 0.86
SP2: Cycling 269 1.14 (0.94, 1.38) 0.19
0.5
1
2
5
Variable
N
TTP: Hazard ratio
p value
age 266 1.00 (0.99, 1.02) 0.63
sex F 122
M 144 0.97 (0.71, 1.34) 0.86
stage 1 69
2 192 1.26 (0.85, 1.85) 0.25
3 5 1.22 (0.28, 5.22) 0.79
grade 0 48
1 150 1.68 (1.04, 2.71) 0.03
2 68 1.41 (0.81, 2.45) 0.22
SP1: Ribosomal 266 0.94 (0.77, 1.16) 0.57
SP2: Cycling 266 1.23 (1.00, 1.50) 0.05
0.5
1
2
5
D
Vascular endothelial
Lymphatic endothelial
Pericyte
Vascular smooth muscle
Cancer-associated fibroblast
Adipocyte
Epithelial (malignant)
Epithelial (non-malignant)
Endocrine
Lymphoid
Myeloid
LP1: Capillary
LP2: Arterial
LP3: Venous
LP4: Lymphatic
Lineage Program 5
Normalized mean
expression in group
0.0
0.5
1.0
